# Weighted Vest Use during Dietary Weight Loss on Bone Health in Older Adults with Obesity

**DOI:** 10.4172/2329-9509.1000210

**Published:** 2017-11-28

**Authors:** Jessica L Kelleher, Daniel P Beavers, Rebecca M Henderson, Dixie Yow, Charlotte Crotts, Jessica Kiel, Barbara J Nicklas, Kristen M Beavers

**Affiliations:** 1 Department of Health and Exercise Science, Wake Forest University, Winston-Salem, NC 27109, USA; 2 Department of Biostatistics, Wake Forest University, Winston-Salem, NC 27157, USA; 3 Section on Gerontology and Geriatric Medicine, Wake Forest School of Medicine, Winston-Salem, NC 27157, USA; 4 Department of Scientific and Clinical Affairs, Medifast Inc., Owings Mills, MD, 21117, USA

**Keywords:** Weight loss, Weighted vest, Obesity, Bone mineral density, Bone turnover

## Abstract

**Background:**

To examine the effects of daily weighted vest use during a dietary weight loss intervention, on (a) hip and spine bone mineral density (aBMD), and (b) biomarkers of bone turnover, in older adults with obesity.

**Methods:**

37 older (70.1 ± 3.0 years) adults with obesity (BMI=35.3 ± 2.9) underwent a 22 week dietary weight loss intervention (1100–1300 kcal/day) with (Diet+Vest; n=20) or without (Diet; n=17) weighted vest use (goal: 10+ h/day; weight added incrementally based on amount of weight lost). Total body weight; DXA-acquired aBMD of the total hip, femoral neck and lumbar spine; and biomarkers of bone turnover (OC, BALP, P1NP, CTX) were measured at baseline and follow up. General linear models, adjusted for baseline values of the outcome and gender, were used to examine intervention effects.

**Results:**

Average weight loss was significant in both groups (−11.2 ± 4.4 kg and −11.0 ± 6.3 kg, Diet+Vest and Diet groups, respectively), with no difference between groups (p=0.91). Average weighted vest use was 6.7 ± 2.2 h/day. No significant changes in aBMD or biomarkers were observed, although trends were noted for total hip aBMD and BALP. Loss in total hip aBMD was greater in the Diet group compared with Diet+Vest (Δ: −18.7 [29.3, −8.1] mg/cm^2^ versus −6.1 [−15.7, 3.5] mg/cm^2^; p=0.08). BALP increased in the Diet+Vest group by 3.8% (Δ: 0.59 [−0.33, 1.50] μg/L) and decreased by −4.6% in the Diet group (Δ: −0.70 [−1.70, 0.31] μg/L, p=0.07).

**Conclusion:**

Weighted vest use during weight loss may attenuate loss of hip aBMD and increase bone formation in older adults with obesity. Further study is warranted.

## Introduction

Recommendation for intentional weight loss in advanced aged individuals (i.e., 65+ years) remains controversial due to weight loss-associated loss of bone mass [1–4] and potential exacerbation of age-related osteoporotic fracture risk [5–8]. One potential strategy to preserve bone health during a diet induced weight loss program is to add weight-bearing exercise. The osteogenic effect of exercise in weight stable older adults is well recognized [9] and is attributed to the increased mechanical stress placed on bone tissue [10]. However, data from randomized controlled trials (RCTs) specifically designed to assess the effect of exercise on bone mass during weight loss are limited, with mixed findings reported [11–13]. Moreover, exercise participation among older adults is strikingly low, with less than 10% of adults over the age of 65 meeting national physical activity guidelines [14]. In fact, compliance may be a primary factor explaining discrepant trial findings [15], and speaks to the larger issue of identification of easily translatable weight loss countermeasures to minimize bone loss.

Preventing reductions in mechanical load via use of a weighted vest may offer an alternative to exercise training to attenuate weight loss-associated bone loss in older adults with obesity. Skeletal tissue is highly responsive to mechanical perturbation [16] and most data show that the magnitude of decline in bone density is proportional to the amount of total weight lost, suggesting that reduced mechanical stress is one mechanism underlying the loss of bone in response to weight reduction. Prior clinical studies of weighted vest use are limited, but do provide support for this concept. For instance, walking while wearing a vest weighted with up to 8% of body mass increases loading of the skeletal system, and thus causes increased bone formation and decreased bone resorption in weight stable adults, when compared to sedentary controls [17]. Similarly, wearing a weighted vest while strength training and stair climbing for one hour per day, three days per week increased femoral neck bone mineral density in older adults, when compared to controls [18]. The effects of weighted vest use during caloric restriction, however, as a means to attenuate weight loss-associated bone loss has not yet been studied.

Therefore, the purpose of this pilot study was to examine daily weighted vest use during a 22 week dietary weight loss intervention in older adults with obesity, and generate preliminary treatment effect estimates on two clinically relevant indicators of bone health and subsequent fracture risk: (a) DXA-acquired regional BMD, and (b) biomarkers of bone turnover.

## Methods

### Study design

This 22 week randomized, controlled pilot trial (http://clinicaltrials.gov; NCT02239939) examined the effect of daily use of a weighted vest during dietary weight loss (Diet+Vest=20), as compared to a diet only control (Diet=17), in older adults with obesity. Primary aims included estimating the variability of treatment effects on regional BMD and biomarkers of bone turnover. Participants were recruited and enrolled based on the following criteria: 1) 65–79 years; 2) sedentary; 3) BMI of 30–40 kg/m^2^; 4) non-smoking; 5) weight stable (<5% weight change in the past 6 months); and 6) without comorbidities for which the intervention was contraindicated. The study was approved by the Wake Forest School of Medicine Institutional Review Board, and all participants provided written informed consent.

## Interventions

### Dietary weight loss intervention

All participants underwent a dietary weight loss intervention targeting 8–10% weight loss. Caloric deficit was achieved through a combination of meal replacements (MR), conventional foods, and weekly group nutrition/behavioral counseling sessions led by a Registered Dietitian (RD). All participants were instructed to follow the Medifast^®^ 4 and 2 and 1 Plan^®^, estimated to provide 1100–1300 cal/day. This meal plan includes a total of 4 MR products per day, with the addition of 2 lean and green meals and 1 healthy snack. The lean and green meals each consisted of 5–7 oz. lean protein, 3 servings of nonstarchy vegetables and up to 2 servings of healthy fat. The healthy snack consisted of one serving of fruit, dairy, or grain. The MR from Medifast^®^ each contained ~90–110 kcal and 11–15 g protein. Daily food logs were collected and reviewed weekly by the RD.

### Weighted vest intervention

Participants randomized to the weighted vest group (Diet+Vest) each received an appropriately sized weighted vest (Hyper Vest PRO^®^, Austin, TX) for the duration of the intervention. The vest fits comfortably under clothing, allowing for full range of motion and movement. Small slots in the vest allow the 1/8th pound weights to be evenly distributed throughout the vest. Participants in this group were asked to wear the vest on a daily basis, progressing to a goal of 10 h/day during the most active part of their day. Initially, no weight was added to the vest (vest weight alone is ~0.5 kg). The vest weight was then incrementally increased weekly according to each participant’s weight loss, to a maximum amount of 15% of the participant’s baseline weight. Participants also kept a daily log to record the time worn, vest weight, and any problems related to the vest use.

## Measures

### Relevant covariates

Baseline demographics such as age, gender, and ethnicity were recorded based on participant self-report. Baseline and follow up weights were measured without shoes or outer garments on a Detecto scale (Detecto, Webb City, MO). Height was measured without shoes using the Heightronic 235D stadiometer (QuickMedical, Issaquah, WA). Baseline height and weight were measured at the first screening visit and were used to calculate baseline body mass index (BMI).

### Intervention process measures

Weekly weights were collected using a Tanita BWB 800 scale (Tanita, Arlington Heights, IL) in order to track the rate of weight loss. Baseline and the average of the two 22 week follow up visit weights measured on a Detecto scale (Detecto, Webb City, MO) were used to determine total amount of weight lost. Percent compliance to the dietary intervention was determined by the RD by calculating the number of calories consumed daily (based on the self-reported food logs) relative to the estimated calories prescribed (~1200). The amount of time spent wearing the vest daily and weight in the vest were collected in self-report weekly logs completed by the Diet+Vest group. This data was used to calculate percent compliance based on 10 hours per day goal in order to determine compliance to the weighted vest protocol. Weight in the vest was recorded weekly by research staff.

### Bone mineral density

Dual-energy X-ray absorptiometry (DXA) (Delphi QDR; Hologic), was used to obtain measures of areal BMD (aBMD) at the total hip, femoral neck, and lumbar spine at baseline and follow up. All scans were performed and analyzed by an ISCD certified DXA technician who evaluated each scan for proper patient positioning and analysis. Coefficients of variation (CV) from repeated measurements are 1.38% for lumbar spine aBMD, 1.21% for total hip aBMD and 1.82% for femoral neck aBMD.

### Biomarkers of bone turnover

Systemic biomarkers of interest include; bone formation markers Osteocalcin (OC), Bone Specific Alkaline Phosphatase (BALP) and Procollagen Type 1 N-Terminal Propeptide (P1NP) and bone resorption marker C-Terminal Telopeptide of Type 1 Collagen (CTX). All blood samples were drawn under standard fasting conditions at baseline (n=37) and follow up (n=33) and stored at −80°C until later analysis.

Both CTX and P1NP were analyzed using enzyme-linked immunosorbent assay (ELISA) kits from Neo Scientific (Cambridge, MA). OC was analyzed using Quantikine ELISA kits from R&D systems (Minneapolis, MN). All samples were run in duplicate. The average of both readings was used for data analysis. BALP analyses were performed at a clinical laboratory (LabCorp, USA) following standard procedures.

### Statistical analysis

Baseline demographic and clinical characteristics were summarized using descriptive measures by group and overall. Treatment effects on bone biomarkers and aBMD were estimated using general linear models both unadjusted and adjusted for baseline values of the outcome and gender. A correlation coefficient was calculated to determine the association of weight added to the vest and the amount of weight lost in the Diet+Vest group. As a pilot study, the study sample size was determined to generate estimates for future power calculations; therefore, all comparisons of treatment efficacy are considered exploratory rather than confirmatory, and a significance level of 0.05 is used throughout. All analyses were performed using SAS v9.4 (SAS Institute, Cary, NC).

## Results

### Baseline characteristics

Thirty-three of the 37 randomly assigned participants (89%) completed the study. The retention of participants was not different between groups (Diet: 88%; Diet+Vest: 90%). Overall participants were aged 70.1 ± 3.0 years, 78.4% were female, 75.7% were white, and baseline BMI was 35.3 ± 2.9 kg/m^2^. No significant group differences were observed for baseline characteristics (Table 1). Data for baseline measures of bone health and associated reference ranges are provided in Table 1. Overall, values of DXA-acquired aBMD and biomarkers of bone turnover did not differ between groups.

### Intervention process measures

Both groups experienced similar and significant weight loss (Diet=−11.2 ± 4.4 kg; 11.9% and Diet+Vest=−11.0 ± 6.3 kg; 10.9%; both p<0.001 compared to baseline), with no difference between groups (p=0.91). Dietary compliance was high and similar between groups, with participants meeting daily caloric recommendations an average of 95.0 ± 9.2% of intervention days. The Diet+Vest group wore the vest for an average of 6.7 ± 2.2 h/day (range of 2.0–9.9 h/day). Overall, participants reported meeting the vest-wear goal of 10 hours/day for 67 ± 22% of the total intervention days. The weight in the vest averaged 6.3 ± 2.5 kg or 7.1 ± 3.0% of baseline body weight, which was strongly correlated to the individual amount of weight lost by participant (r=0.76).

### Treatment effects on bone density and turnover

**Figure 1:**
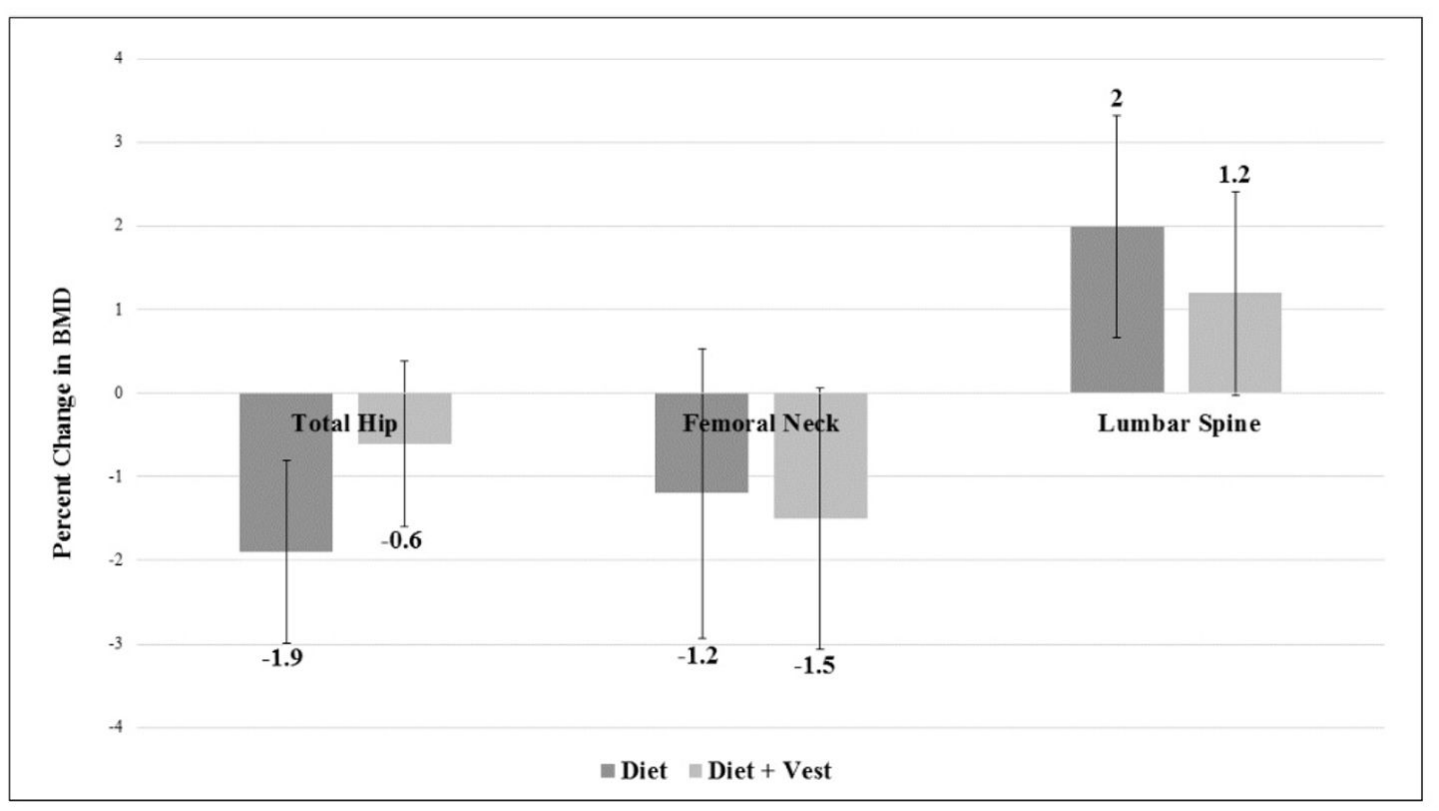
22 week treatment effects on DXA-acquired regional BMD, adjusted for baseline values of the outcome and gender and presented as means (95% CI).

Data regarding 22 week treatment effects on regional DXA acquired aBMD, adjusted for baseline values of outcome and gender, can be viewed in Table 2 and are presented as means (95% CI). No significant differences were seen between groups in aBMD at the total hip, femoral neck or lumbar spine. However, trends toward significance were noted for changes in total hip aBMD, in that the Diet group experienced three times greater decreases as compared to the Diet +Vest group (−18.7 [−29.3, −8.1] mg/cm^2^ versus −6.1 [−15.7, 3.5] mg/cm^2^; p=0.08). The trend toward significance can also be seen in percent change in total hip aBMD, in that the Diet group saw a 1.9% decrease, while the Diet+Vest group experienced only a 0.6% decrease (p=0.08) (Figure 1).

Data for 22 week treatment effects on biomarkers of bone turnover adjusted for baseline values of outcome and gender can be viewed in Table 2 and are presented as means (95% CI). No significant differences between groups were noted for changes in OC, BALP, P1NP and CTX. Trends towards significance were noted for bone formation marker BALP, in that the Diet+Vest group experienced increases, while the Diet group decreased (0.59 [−0.33, 1.50] μg/L versus −0.70 [−1.70, 0.31] μg/L; p=0.07). Additionally, while not significant, biomarkers of formation OC and P1NP appeared to increase or be attenuated in the Diet+Vest group compared to the Diet group (OC: 0.63 [−3.77, 5.03] ng/mL versus −0.07 [−4.89, 4.76] ng/mL; p=0.83, P1NP:−0.06 [−0.47, 0.35] ng/L versus −0.24 [−0.71, 0.24] ng/L; p=0.57).

**Figure 2:**
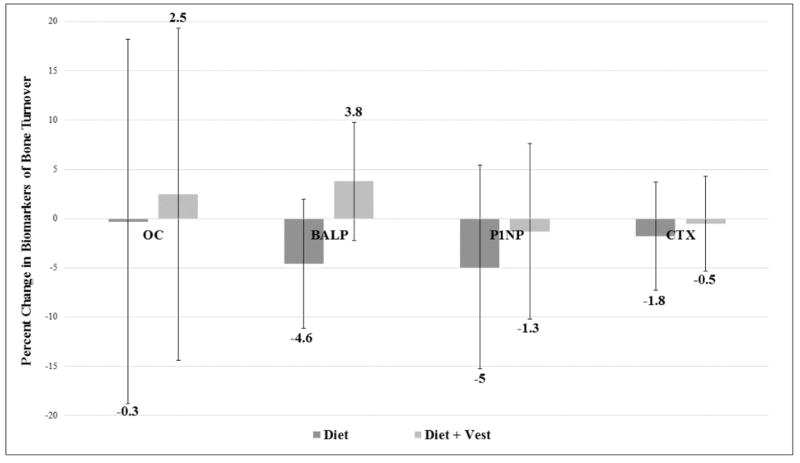
22 week treatment effects on biomarkers of bone turnover, adjusted for baseline values of the outcome and gender and presented as means (95% CI).

The trend toward significance for biomarker BALP is more clearly depicted when expressed as percent change, in that the Diet+Vest group experienced a 3.8% increase, while the Diet group saw a 4.6% decrease (p=0.07) (Figure 2). Stratification of aBMD and biomarker treatment effects by gender are presented in supplementary Tables 1 and 2, with overall results largely aligning with the female subset due to the disproportionate about of women (78%) in the study sample.

## Discussion

The use of a weighted vest to mimic gravitational loading for at least 1/4th of a day over a 22 week period, coupled with a dietary intervention protocol inducing an average weight loss among both groups of 11.3%, resulted in marginally attenuated losses in total hip aBMD in the Diet+Vest group (−6.1 mg/cm^2^ vs. −18.7 mg/cm^2^; p=0.08) and increased BALP (0.59 μg/L vs. −0.70 μg/L or +3.8% vs. −4.6%; p=0.07) compared to diet only. These provocative findings warrant replication from a larger, adequately powered trial.

To date, no study has examined the combination of weighted vest use during dietary weight loss on bone health, although several studies have examined components individually. Behavioral-based weight loss interventions (yielding 7–10% weight loss), for example, consistently result in a loss of hip aBMD to the order of 0.010 to 0.015 g/cm^2^ [2]. This treatment effect is on par with what we observed in the Diet group (i.e., 0.019 g/cm^2^) and although smaller than what is considered clinically meaningful for fracture risk prediction [19], it is larger than what might be expected annually from advanced age alone (0.002–0.006 g/cm^2^/year) [20]. Weighted vest use in weight stable adults has been shown to attenuate age-related loss in hip aBMD by around 0.025 g/cm^2^, when compared to active controls [21]. Thus, original findings presented here showing modestly attenuated loss in hip aBMD by 0.013 g/cm^2^ when weighted vest use is coupled with weight loss, confirm and extend prior literature demonstrating the osteogenic potential of weighted vest use and may signal long term clinical significance.

Similarly, the effect of diet induced weight loss has been shown to affect biomarkers of bone turnover, in particular, those of bone resorption. Key findings from a recent meta-analysis show significant increases in both CTX and NTX of 4.72 nmol/L (95% CI, 2.12 to 7.30 nmol/L) and 3.70 nmol/L (95% CI, 0.90 to 6.50 nmol/L) for weight loss studies lasting 2 or 3 months in duration. This indicates an early effect of diet induced weight loss to promote bone resorption that may have been missed in the present study due to biomarker measurement occurring only at baseline and 22 week follow up. Weighted vest use in weight stable adults has been shown to affect biomarkers of bone formation. A six week RCT conducted by Roghani et al. [17] saw increases in BALP of 7.3% (p=0.05) in the exercise and weighted vest group and 10.3% (p=0.03) in the exercise only group; compared to their sedentary control group who experienced a 1.9% decrease. This is similar to the favorable effects shown in the present study, in that the Diet+Vest group experienced a 3.8% increase in BALP, while the Diet group saw a 4.6% decrease (p=0.07).

Although not specifically designed to examine the effect of weighted vest use during intentional weight loss, it is worth discussing the present findings in light of the lone RCT of weighted vest use where weight loss was achieved through exercise alone [18]. In 2003, Jessup et al. [18] randomized healthy, Caucasian women to participate in a 32 week exercise program while wearing a weighted vest (up to 10% of baseline body weight) or to a sedentary control group. Women wearing the weighted vest lost 5% of their body weight, yet increased femoral neck aBMD by 1.7%, while sedentary controls decreased by 0.4% (p=0.02). Although participants in the Diet+Vest group of the present study did not experience increases in aBMD, total hip aBMD loss was attenuated in comparison to the Diet group (−0.6% versus- 1.9%, p=0.08), which supports an osteogenic role of weighted vest use. This minor discordance may be due to absolute weight loss differences (5% loss [18] versus 11% loss in the present study) and fundamental differences between exercise-induced and diet-induced weight loss [22].

The major strengths of this study are the novelty of the intervention design and measurement of aBMD at various sites and four biomarkers of bone turnover in order to provide a comprehensive assessment of the state of bone remodeling in response to treatment. The most notable limitation of this study is the small, predominantly female sample, which did not allow for sufficient power to detect statistically significant treatment effects. Additionally, we did not observe consistent treatment effects across bone regions and biomarkers, calling into question the robustness of our findings. That being said, the lumbar spine region is notorious for measurement error, especially in the context of obesity and osteoarthritis [2] and the femoral neck - having a smaller area - is more subject to variability; thus it is not surprising that we were unable to detect differences at these locations in our small study sample. Changes to bone biomarker CTX also differed from that experienced in previous studies, which may have been due to the timing of blood sampling [2], although the directionality of treatment effects in BALP, OC and P1NP were in accordance with our hypothesis.

In sum, novel findings from this study indicate the potential efficacy of weighted vest use during intentional weight loss to modestly attenuate loss in total hip aBMD while increasing bone formation. Further research is needed in an adequately powered sample to confirm pilot findings, and to investigate the long-term effect of weighted vest use to minimize weight loss-associated bone loss. Future areas of inquiry also include direct comparison of the weighted vest therapy during weight loss to more mainstream osteogenic strategies, such as resistance training or pharmacotherapy.

## Figures and Tables

**Table 1:** Participant baseline characteristics (Presented as means ± SD or n (%). Note: DXA-acquired aBMD: Duel Energy X-ray Absorptiometry acquired areal Bone Mineral Density; OC: Osteocalcin; BALP: Bone Specific Alkaline Phosphatase; P1NP: Procollagen Type 1 N-Terminal Propeptide; CTX: C-Terminal Telopeptide of Type 1 Collagen)

	Diet only	Diet + Vest	Reference Ranges
	n=17	n=20	
Age (years)	69.9 ± 2.6	70.3 ± 3.4	
Female, n (%)	14 (82.4)	15 (75.0)	
White, n (%)	13 (76.5)	16 (80.0)	
Weight (kg)	93.8 ± 11.5	99.4 ± 10.3	
Body Mass Index (kg/m^2^)	35.3 ± 3.0	35.3 ± 2.8	
DXA-acquired aBMD (g/cm^2^)			0.85–1.25
Total Hip	0.94 ± 0.14	0.98 ± 0.13	
Femoral Neck	0.74 ± 0.10	0.81 ± 0.12	
Lumbar Spine	1.11 ± 0.19	1.16 ± 0.19	
Biomarkers of Bone Turnover			
OC (ng/mL)	24.7 ± 15.3	27.3 ± 21.5	0–64
BALP (μg/L)	15.5 ± 5.6	15.1 ± 5.2	7.4–25.4
P1NP (ng/mL)	4.3 ± 2.4	4.8 ± 1.4	1.2–3.9
CTX (ng/mL)	6.8 ± 2.0	5.7 ± 2.6	0.8–5.3

**Table 2:** 22 week treatment effects on DXA-acquired regional BMD and biomarkers of bone turnover (Adjusted for Baseline Values of the Outcome and Gender) (Note: aBMD: areal Bone Mineral Density; OC: Osteocalcin; BALP: Bone Specific Alkaline Phosphatase; P1NP: Procollagen Type 1 N-Terminal Propeptide; CTX: C-Terminal Telopeptide of Type 1 Collagen).

	Diet only	Diet+Vest	*p*-value
	Mean (95% CI)	Mean (95% CI)	
**Δ DXA-acquired aBMD (mg/cm^2^)**			
Total Hip	−18.7 (−29.3, −8.1)	−6.1 (−15.7, 3.5)	0.08
Femoral Neck	−9.8 (−23.3, 3.7)	−11.9 (−24.1, 0.3)	0.82
Lumbar Spine	22.9 (7.7, 38.0)	13.7 (−0.2, 27.5)	0.37
**Δ Biomarkers of Bone Turnover**			
OC (ng/mL)	−0.07 (−4.89, 4.76)	0.63 (−3.77, 5.03)	0.83
BALP (μg/L)	−0.70 (−1.70, 0.31)	0.59 (−0.33, 1.50)	0.07
P1NP (ng/mL)	−0.24 (−0.71, 0.24)	−0.06 (−0.47, 0.35)	0.57
CTX (ng/mL)	−0.11 (−0.45, 0.23)	−0.03 (−0.33, 0.27)	0.73
